# Phylogenomics and Diversification of the Schistosomatidae Based on Targeted Sequence Capture of Ultra-Conserved Elements

**DOI:** 10.3390/pathogens11070769

**Published:** 2022-07-05

**Authors:** Erika T. Ebbs, Eric S. Loker, Lijing Bu, Sean A. Locke, Vasyl V. Tkach, Ramesh Devkota, Veronica R. Flores, Hudson A. Pinto, Sara V. Brant

**Affiliations:** 1 Department of Biology, Purchase College, The State University of New York, Purchase, NY 10577, USA; 2 Center for Evolutionary and Theoretical Immunology, Department of Biology, Museum of Southwestern Biology Parasite Division, University of New Mexico, Albuquerque, NM 87131, USA; esloker@unm.edu (E.S.L.); lijing@unm.edu (L.B.); sbrant@unm.edu (S.V.B.); 3 Department of Biology, University of Puerto Rico at Mayagüez, Box 9000, Mayagüez 00681-9000, Puerto Rico; sean.locke@upr.edu; 4 Grand Forks Department of Biology, University of North Dakota, Grand Forks, ND 58202, USA; vasyl.tkach@und.edu; 5 Vance Granville Community College, Henderson, NC 27536, USA; devkotar@vgcc.edu; 6 Laboratorio de Parasitología, INIBIOMA (CONICET-Universidad Nacional del Comahue), Quintral 1250, San Carlos de Bariloche 8400, Argentina; veronicaroxanaflores@gmail.com; 7 Department of Parasitology, Institute of Biological Science, Universidade Federal de Minas Gerais, Belo Horizonte 31270-901, Brazil; hudsonalves13@icb.ufmg.br

**Keywords:** schistosomatid, blood fluke, UCE, parasite phylogenomics, sequence capture, diversification, host-switching, museum collections, voucher

## Abstract

Schistosomatidae Stiles and Hassall 1898 is a medically significant family of digenetic trematodes (Trematoda: Digenea), members of which infect mammals or birds as definitive hosts and aquatic or amphibious gastropods as intermediate hosts. Currently, there are 17 named genera, for many of which evolutionary interrelationships remain unresolved. The lack of a resolved phylogeny has encumbered our understanding of schistosomatid evolution, specifically patterns of host-use and the role of host-switching in diversification. Here, we used targeted sequence capture of ultra-conserved elements (UCEs) from representatives of 13 of the 17 named genera and 11 undescribed lineages that are presumed to represent either novel genera or species to generate a phylogenomic dataset for the estimation of schistosomatid interrelationships. This study represents the largest phylogenetic effort within the Schistosomatidae in both the number of loci and breadth of taxon sampling. We present a near-comprehensive family-level phylogeny providing resolution to several clades of long-standing uncertainty within Schistosomatidae, including resolution for the placement of the North American mammalian schistosomes, implying a second separate capture of mammalian hosts. Additionally, we present evidence for the placement of *Macrobilharzia* at the base of the *Schistosoma* + *Bivitellobilharzia* radiation. Patterns of definitive and intermediate host use and a strong role for intermediate host-switching are discussed relative to schistosomatid diversification.

## 1. Introduction

An informed understanding of diversification is lacking for most multi-host helminth groups [1,2]. In fact, few such groups have been sampled sufficiently to reconstruct a reliable, well-resolved phylogeny, which is necessary to understand parasite evolution. Elucidating helminth diversification has practical implications for knowledge and the management of human and wildlife helminths, issues relating to host-competency [3], potential for host-switching [4,5,6,7], host associations [8,9,10], character evolution [11], and cladogenesis [12,13]. Among digenetic trematodes, the Schistosomatidae Stiles and Hassall 1898 is a diverse family with its members infecting birds or mammals (definitive hosts) and aquatic or amphibious gastropods (intermediate hosts). The Schistosomatidae have garnered substantial interest among parasitologists, due to the medical and veterinary importance of its members. Three species within the genus *Schistosoma* Weinland, 1858 are the major etiological agents of human schistosomiasis, one of the world’s most recalcitrant neglected tropical diseases still infecting over 250 million people globally [14,15]. Moreover, non-human schistosomatids are involved in human cercarial dermatitis (swimmer’s itch), a re-emerging zoonotic disease [16,17].

Among digeneans, schistosomatids have unusual characteristics, the most notable being that they are exclusively dioecious (usually with marked sexual dimorphism) parasites of endotherms (birds and mammals), which are collective attributes not shared with any other digenean family [18,19]. Schistosomatids have evolved heterogametic (ZW) sex chromosomes [18], which function in sex determination. Schistosomatids possess other interesting reproductive strategies [20,21] and are among the few digenean lineages in which adults reside outside the alimentary canal of their vertebrate definitive hosts. They also lack a second intermediate host in their life cycles. Decades of sequencing of the DNA from schistosomatids from their definitive and intermediate hosts (e.g., see other papers in this volume) coupled with the use of well-vouchered museum specimens have shed light on the previously under-recognized diversity of schistosomatids, especially for avian-infecting species, including species responsible for human cercarial dermatitis. This effort has provided a foundation to develop a robust evolutionary framework to address schistosomatid diversity and diversification worldwide.

Despite advances, our understanding of schistosomatid interrelationships, patterns of host-use, and character evolution remains incomplete [20,22,23,24]. Progress has been constrained by a lack of informative morphological characteristics and adequate adult parasite material for description. The inclusion of molecular analyses [25,26,27] has greatly expanded efforts to explain schistosomatid diversity, particularly for avian-infecting genera, which have been proven to contain cryptic diversity [10,28]. Although these and other studies have made considerable strides, deeper nodes pertaining to the interrelationships between genera remain unresolved and often provide conflicting phylogenetic signals. This is likely because molecular phylogenies have been based on few loci, primarily markers within the ribosomal RNA operon (*28*S, *18*S, ITS1, and 2) and a single mitochondrial gene (*cox*1).

Currently, within the Schistosomatidae, there are 17 named genera and over 130 named species [21]. Based on the sampling of larval schistosomatids, this is likely an underestimate [8,29,30,31,32,33,34,35] of both genera and species. Historically, Schistosomatidae was divided into three subfamilies [24,36], primarily based on the morphology of adult worms. Schistosomatinae Stiles and Hassall 1898 includes *Austrobilharzia*, *Bivitellobilharzia*, *Heterobilharzia*, *Macrobilharzia Ornithobilharzia*, *Schistosomatium,* and *Schistosoma*. Gigantobilharziinae Mehra 1940 comprises *Gigantobilharzia* and *Dendritobilharzia*. Bilharziellinae Price 1929 includes *Bilharziella*, *Trichobilharzia*, *Jilinobilharzia*, *Allobilharzia,* and *Anserobilharzia*. A fourth subfamily, Griphobilharziinae Platt, Blair, Purdie & Melville, 1991, containing a single species *Griphobilharzia amoena*, has been recognized, but sequence-based data places this species within the Spirorchiidae [36]. Molecular phylogenies have not supported these subfamily designations [21,25,26,27,31,34,37,38,39], and the most appropriate subfamily classifications remain unclear.

As illustrated by the conflicting results from prior studies (Table 1), the continued sequencing of 500–1500 bp markers seems unlikely to resolve deeper schistosomatid relationships. New methodologies are needed to resolve the following pivotal nodes and groups, and thus better characterize diversification, evolution of host-use, host switches and divergence times within Schistosomatidae:

1. **AO Clade:** Marine avian genera, *Austrobilharzia* and *Ornithobilharzia* (AO clade), are often recovered as well-supported sister genera [36,39,40], and both are considered globally distributed. In most of the studies over the last 10 years, the AO clade typically acts as a sister group to the remainder of the schistosomatids.
2. **SB Clade:** Species of the Afro-Eurasian mammalian clade (*Schistosoma* + *Bivitellobilharzia*, or the SB clade) are found in tropical and sub-tropical latitudes. *Schistosoma* and *Bivitellobilharzia* are considered probable sister genera, but the use of traditional loci often does not statistically support this grouping. A better understanding of relationships within the SB clade and its placement within Schistosomatidae is essential to understanding the evolution of human schistosomatids.
3. ***Macrobilharzia*:***Macrobilharzia* is a monotypic genus with species that infect *Anhinga anhinga* in the Americas and has failed to group consistently with other schistosomatid lineages. Some rRNA phylogenies suggest an (unsupported) affinity with the SB clade, suggesting the possibility of the SB clade having had Afro-Eurasian avian-infecting ancestors. Cercariae (Schistosomatidae-W688) recovered from the freshwater snail *Indoplanorbis exustus* in Nepal [41] represent an otherwise undescribed, but well-supported, sister lineage to *M. macrobilharzia*.
4. **DAS Clade:** The derived avian–schistosomatid (DAS) clade includes the majority of avian-infecting genera (*Anserobilharzia*, *Allobilharzia*, *Bilharziella*, *Dendritobilharzia*, *Gigantobilharzia*, *Marinabilharzia*, *Nasusbilharzia*, *Riverabilharzia*, *Trichobilharzia*) and several yet-to-be described genera. The monophyly of DAS is consistently supported (Table 1, [39,40]), though its placement as a sister to the SB clade or North American mammalian schistosomatids is unclear. The relationships within the DAS clade are largely unresolved.
5. **HS Clade:***Heterobilharzia* and *Schistosomatium* (HS clade) are both monotypic genera whose species infect North American mammals and form a well-supported clade in most studies (Table 1). Phylogenies based on oft-used loci have provided weak support for the placement of the HS clade as a sister to the DAS clade (see Table 1). Phylogenetic placement of the HS clade has significant implications for understanding the role of host shifts and biogeography of the Schistosomatidae.

The conflict among these pivotal nodes (Table 1) might be explained by widespread incomplete lineage sorting (ILS) within the Schistosomatidae, as suggested by previous authors [21,27]. Resolving relationships in this context requires increasing the number of characters analyzed, ideally through augmenting the number of independently evolving loci, as well as more complete taxon sampling [42,43,44]. Recent analyses of whole mitogenomes for species of *Schistosoma* [45] have yielded increased resolution, however this approach is unlikely to yield similar results when applied to the Schistosomatidae, as mitochondrial loci evolve in concert. Moreover, among digeneans, whole mitochondrial genomes present evolutionary trajectories that differ strikingly at deeper levels from nuclear genomes [46,47], suggesting a need for caution. Recent developments in phylogenomic methods have proven effective in resolving clades where ILS was suspected [48,49]. One such method uses the targeted sequence capture of ultra-conserved elements (UCEs) [50] to obtain data from hundreds to thousands of independent nuclear loci for phylogenomic analysis. This method requires a panel of probes that target UCE loci and less conserved flanking regions in the group of interest, which are sequenced using next-generation methods, producing alignments of thousands to millions of bases that can resolve divergence points across different time scales [50]. The use of UCEs as phylogenomic markers has been successful at resolving rapid radiations within vertebrates [51,52,53,54] as well as relationships within an increasing number of invertebrate groups [55,56,57,58]. Within Digenea, the only application of this approach was characterized by a relatively low number of loci (517 UCEs in [47]).

The present study applied a sequence capture approach to generate UCE loci for vouchered schistosomatid specimens collected over a 20+ year period, to generate a more fully resolved phylogeny of Schistosomatidae, to understand patterns of host-switching, character evolution and more broadly, diversification. This more resolved phylogeny will enable the generation of more targeted phylogenetic hypotheses regarding schistosomatid diversification, and can be applied to address questions such as: have specific traits aided schistosomatid diversification? How common is intermediate and definitive host-switching within Schistosomatidae? Did the major schistosomatid clades radiate simultaneously? Additionally, a resolved phylogeny will provide direction for future targeted collection efforts and will help characterize the timing of schistosomatid lineage formation.

## 2. Results

### 2.1. UCE Enrichment and Sequencing

Targeted sequence capture of UCE loci was performed on 39 schistosomatid samples (Table 2). Multiplexed sequencing of enriched libraries resulted in an average 6,939,218 (23,988–24,435,956) reads per sample, with an average sequencing depth of 350× (58–1001×). An average of 44,060 (12–442,043) contigs with a mean length of 325 bp was assembled. On average, we recovered ~500, ~1500, ~884, and ~850 UCE loci in R1, R2, R3, and R4, respectively (Table 3). This is a lower recovery rate relative to the published sequence capture datasets of vertebrates [51,52] and the few existing studies on invertebrates [51], which is likely related to the low amounts of starting DNA (Table 3, [59]).

### 2.2. Phylogenetic Reconstruction

#### 2.2.1. Supermatrix Alignment

**Figure 1:**
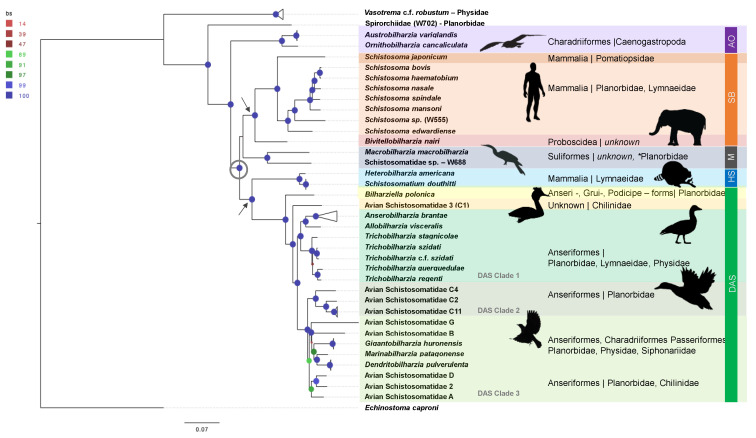
RAxML analyses of concatenated UCE loci. Phylogeny was estimated using the maximum likelihood method using a GTRGAMMA + I model, based on 554 UCE loci. Node circles indicate the bootstrap support value, corresponding to the legend in the top left corner. The tree was edited in Fig Tree v 1.4. The gray open circle denotes the major divergence of the derived schistosomatids. The two gray arrows denote the two major derived nodes leading to two simultaneous and independent radiations, one predominantly in birds the other predominantly in mammals. Spirorchiidae samples fall into two clades concordant with the results of Bullard et al. [61], with a South American species recovered from a freshwater turtle, as sister to Schistosomatidae.

Thirteen unique alignments, ranging from being 82–36.4% complete, were analyzed and their likelihood scores were calculated in RAxML (Supplementary File S2, [60]), to determine an appropriate level of data completeness for subsequent analyses. The UCE supermatrix contained taxa and loci, where; (1) ≥70% of taxa were shared for a given locus, and (2) of the remaining loci (nloci = 554), the percentage of nucleotide coverage was ≥70% per locus (Supplementary File S3). The lowest nucleotide coverage per sample, averaged over 554 loci, occurred in W333 (*Anserobilharzia*) and C1 (Avian Schistosomatidae lineage 3). Nucleotide coverage was high in taxa of key interest such as *Macrobilharzia macrobilharzia* (81% of 554 loci)*,* Schistosomatidae sp. W688 (84%), *Heterobilharzia americana* (93%), and *Bivitellobilharzia nairi* (73%) (Figure 1, Supplementary File S3).

#### 2.2.2. Phylogenies

**Figure 2:**
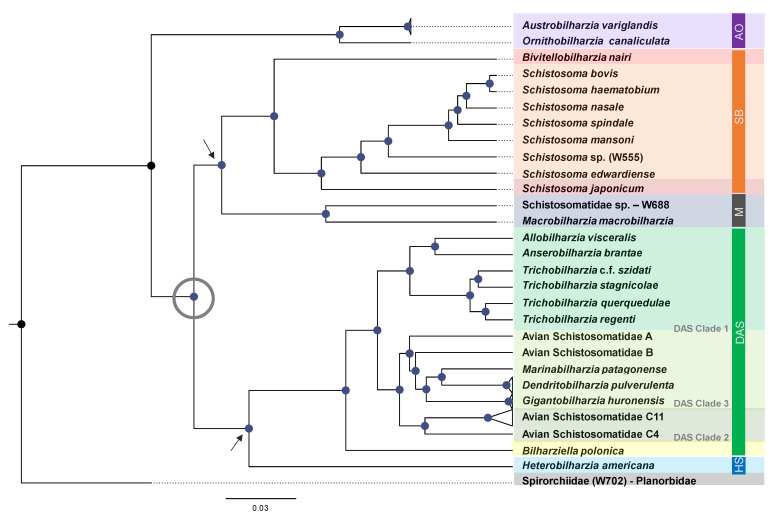
Bayesian inference analysis preformed in BEAST 2 of 85 concatenated Z-chromosome (UCE) loci. Blue node circles indicate posterior probability values of 1, black circles indicate 0.99. Maximum likelihood (RAxML) analysis provided concordant support values with the following exceptions: bootstrap support for (SB + M) = 66%; ((SB + M) (HS + DAS)) = 93%; (*Trichobilharzia* c.f. *szidati* + *T. stagnicolae*) *=* 95%. SB = *Schistosoma + Bivitellobilharzia;* M = *Macrobilharzia +* Schistosomatidae sp. W688; HS = *Heterobilharzia* + *Schistosomatium;* DAS = *Anserobilharzia*, *Allobilharzia*, *Trichobilharzia*, *Dendritobilharzia*, *Gigantobilharzia*, *Bilharziella* and all sampled Avian Schistosomatidae sp. Tree was edited in Fig Tree v 1.4. The gray open circle denotes the major divergence of the derived schistosomatids. The two gray arrows denote the two major derived nodes leading to two simultaneous and independent radiations, one predominantly in birds, the other predominantly in mammals.

Across all phylogenies reconstructed (Figure 1 and Figure 2) and methods of phylogenetic inference (ML and BI), the resulting topologies converged to support the monophyly of the AO, SB, M, HS, and DAS clades, and their position within the Schistosomatidae. The AO clade was grouped as a sister to the rest of the Schistosomatidae, suggesting an early diverging or ancestral position. The remaining schistosomatids will be referred to as “derived” taxa, relative to the AO clade. Among the derived taxa two primary clades formed, (1) SB + M form a clade, with moderate (Figure 1) to strong (Figure 2) support values. The M clade contains strong support for *Macrobilharzia* as a sister genus to Schistosomatidae sp. W688. Within the SB clade, there is strong support for *Bivitellobilharzia* as a sister to *Schistosoma*, and within *Schistosoma* there is strong support for the main species groups [62,63,64] that was recovered. (2) HS + DAS form a clade with strong support. *Bilharziella polonica* was found to be basal relative to the DAS clade.

All recovered loci were mapped to their presumptive chromosomal location within the *Schistosoma mansoni* genome. Of 139 UCE loci that were mapped to the Z-chromosome (Supplementary File S4), 85 were recovered for ≥70% of the taxa (*n* = 35) and used for phylogenetic reconstruction (Figure 2). Loci were represented in 56–90% of the taxa. Moderate Z-chromosome UCE loci representation was obtained in pivotal taxa such as *Macrobilharzia macrobilharzia* (63% of 139 loci)*,* Schistosomatidae sp. W688 (62%), *Heterobilharzia americana* (68%), and *Bivitellobilharzia nairi* (60%).

Both the maximum likelihood and Bayesian inference analyses of Z-chromosome UCE loci yielded topologies congruent with the analysis of genome-wide loci (Figure 1 and Figure 2). In analyses of the Z-chromosome UCE loci, however, nodes across a range of divergence times were better resolved than in the analyses of genome-wide loci. This increase in resolution was particularly pronounced in nodes with a bootstrap support of < 100 or a posterior probability of < 1 in the analysis of genome-wide loci. Bayesian inference of Z-chromosome UCEs resolved all pivotal nodes (Table 1), including strong support for the position of *Macrobilharzia* + Schistosomatidae sp. W688 at the base of the SB clade. The corresponding maximum likelihood analysis of Z-chromosome UCEs also placed *Macrobilharzia* + Schistosomatidae sp. W688 at the base of the SB clade, but with weak support (66% of bootstrap replicates).

### 2.3. UCE Loci Sequence Similarity among Blood Flukes

This study included four non-schistosomatid blood fluke taxa (Table 2): three members of Spirorchiidae (W411, W702 and PS) and one Aporocotylidae (W962, not included in phylogenetic analyses). Within the total supermatrix dataset of 554 loci, 33% (W411), 40 (PS) and 61% (W702) loci were recovered from these samples and were found to be phylogenetically informative. Although this varying ‘completeness’ is not necessarily a function of phylogenetic relatedness, it shows the recovery of a reasonable number of non-schistosomatid loci using a *Schistosoma*-derived bait set (Supplementary File S1). Additionally, a randomly selected (though uniform across samples) subset of 25 loci from Spirorchiidae (W411, W702 and PS) and Aporocotylidae (W962) were on average 82.3% (79.50–85.13%) similar to selected Schistosomatidae (Table 4), with about 3.9% greater similarity in Spirorchiidae than Aporocotylidae.

## 3. Discussion

This is the first study to apply reduced representation phylogenomic methods to address the interrelationships and patterns of diversification within the Schistosomatidae. We reconstructed and compared phylogenies based on 554 nuclear UCE loci (4,780,079 bases). We generated the first phylogenetic tree, for any helminth group, based on Z-chromosome-specific loci (sex chromosome). Based on material available from extant species, our results suggest that schistosomatids first appeared in marine birds and gastropods. They later colonized freshwater snails and both birds and mammals associated with freshwater. Two separate acquisitions of mammalian hosts are supported. From within a diverse freshwater-based lineage of avian schistosomatids, some representatives have secondarily colonized marine gastropod and avian hosts.

Past studies using few or single loci to reconstruct the generic relationships within Schistosomatidae often failed to resolve deeper nodes, particularly those significant to diversification by host switching. Herein, we report the resolution of several pivotal nodes and thus an improved understanding of schistosomatid diversification, mainly in the context of intermediate host use. Relative to other taxa [48,58,65], we noted the reduced recovery of UCE loci, similar to what was found in with the only other UCE digenean phylogeny [47], but a resolution of pivotal nodes was achieved in our study. Below we discuss how these results offer insight into the timing of schistosomatid diversification events and host use.

### 3.1. Resolution of Pivotal Nodes and Host Divergences

Resolved phylogenetic trees are necessary to estimate the timing of diversification events. However, without available fossil data, no primary calibration points exist to estimate divergence times for schistosomatids or other helminths. Parasitologists often use host divergence data to derive secondary calibration points [2,66], but this assumes that parasites have evolved within the bounds of particular hosts. This assumption might be sound where parasites and hosts show evidence of co-phylogeny as the result of a long co-evolutionary history [67,68,69]. However, co-cladogenesis among multi-host parasites appears to be uncommon [1,2,70], particularly in groups with evident switching among distantly related hosts, which erodes co-evolutionary signatures [71]. Using the timing of host divergence as a proxy for parasite divergence calibration therefore requires caution. With this in mind, discussed below are hypothesized host-divergence dates at pivotal nodes which may provide some hard bounds for particular nodes within the Schistosomatidae.

#### 3.1.1. AO Clade

All UCE analyses support the AO clade as a sister to the rest of the Schistosomatidae, suggesting an early diverging position. Members of *Austrobilharzia* and *Ornithobilharzia* infect marine birds and snails. Schistosomatids have been hypothesized to have first diverged from spirorchiids (Spirorchiidae), modern day parasites of marine or freshwater turtles, which are the well supported sister clade to Schistosomatidae [18,27,72]. Extant turtle lineages are estimated to have originated during the mid-late Triassic (240–200 mya, [73]), which may provide an upper limit for the origins of the Schistosomatidae. One of the hallmarks of schistosomatid biology is their adoption of fully intravascular habitats in endotherms, including adult worms living in the liver, portal vein and mesenteric veins where nutrient levels are high. Dioecy is viewed as a consequence of the need to have a muscular male to move against the prevailing flow of blood in the portal system and a slender-bodied female to deposit eggs in the intestinal wall. These essential features may have their origins in partially endothermic turtles such as leatherbacks, but may have flourished in fully endothermic hosts, including theropod dinosaurs and/or their descendants, birds. The closest relatives we know of for these proto-schistosomatids are members of the AO clade found in marine charadriiform birds (Figure 1) and marine caenogastropods (e.g., Potamididae, Batillariidae, Nassariidae, Littorinidae) [21]. The snail family Nassariidae is estimated to have diverged roughly 120 mya [74], while Charadriiformes are thought to have diverged between 23–16 mya [75]. Schistosomatids later colonized freshwater aquatic or amphibious habitats and diverged in the two major clades we see today, SB+M and DAS+HS.

#### 3.1.2. Hypothesized Early Gastropod Host Use Prior to the (M + SB) and (HS +DAS) Split

**Figure 3:**
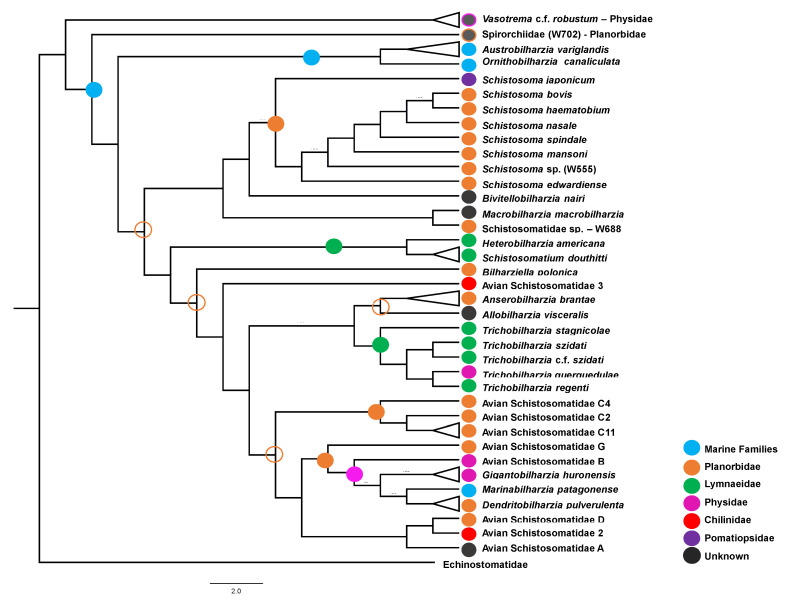
Hypothesized ancestral intermediate host use and host-switching events within the Schistosomatidae. Circles, colored corresponding to the legend, representing hypothesized intermediate host use, were mapped onto the Figure 1 phylogeny. Open circles represent less certainty relative to solid circles.

Some evidence from contemporary gastropod host use among schistosomatids suggests that prior to the (M +SB) and (HS + DAS) split, the prevailing gastropod hosts may have been snails in the family Planorbidae (Figure 3). In support of this, (1) although natural snail hosts are unknown for *Bivitellobilharzia*, experimental hosts were found to be planorbids [76]; (2) planorbids are prominently (but not exclusively) represented as intermediate hosts among *Schistosoma* species; (3) at least one representative of the M clade is hosted by a planorbid (Schistosomatidae sp. W688) [41]; (4) the earliest known diverging member of the DAS clade, *Bilharziella polonica*, has a planorbid snail host, as do many other members of the this clade [21]. A role for lymnaeid snails in hosting ancestral schistosomatids should not be discounted either: (1) a few species of *Schistosoma* use lymnaeid snail hosts; (2) the HS clade use exclusively lymnaeids; (3) they are prominently represented as snail hosts in the DAS clade. Caenogastropods too deserve consideration, as snail hosts prior to the split between the SB + M and DAS + HS clades, insofar as all members of the early diverging *Schistosoma japonicum* species group utilized Pomatiopsidae snails. Davis [77] suggested that proto-*Schistosoma* had co-diversified with amphibious caenogastropods.

#### 3.1.3. SB Clade

Branch lengths in the phylogeny (Figure 1) suggest that the SB + M and DAS + HS clades may have radiated simultaneously. The SB clade represents a substantial radiation within mammals (28 species). Contemporary members of the SB clade infect a broad range of mammalian groups: Bovidae, Canidae, Elephantidae, Felidae, Hippopotamidae, Hominidae, Rhinocerotidae, Suidae, and Rodentia. Relationships among members of the SB clade have been largely resolved [41,64,78]. *Schistosoma* and *Bivitellobilharzia* have been considered to be sister genera despite conflicting phylogenies (Table 1), and all UCE datasets support the monophyly of Afro-Eurasian mammalian schistosomatids. Snyder and Loker [25] hypothesized that *Schistosoma* originated in the mid-Miocene (16–11.6 mya), based largely on estimates by Davis [77] using fossil data to estimate the divergence of Pomatiopsidae snails, hosts to the extant *S. japonicum* clade. The suspected role of this snail family in the diversification of *Schistosoma* led to the use of mt DNA to estimate pomatiopsid divergence between 12–5 mya [79]. Going forward, the identification of the natural intermediate hosts of *Bivitellobilharzia* and *Macrobilharzia* represents important collection goals, as this will help to evaluate the likelihood of the various scenarios highlighted above—planorbid, lymnaeid or pomatiopsid species as ancestral hosts for proto-*Schistosoma*, with possible attendant changes in the timing of molecular divergence among schistosomatids [45].

#### 3.1.4. *Macrobilharzia* (M Clade)

Although branch support values were higher for the Z-chromosome phylogeny (Figure 2) than the concatenated UCE phylogeny (Figure 1), both support an affinity between the M and SB clades (Figure 1 and Figure 2). This suggests a derived rather than an early branching position for the M clade within the Schistosomatidae. Large ribosomal subunit (28S) phylogenies [21,39,41] place *Macrobilharzia* at the base of the SB + DAS clades, thus diverging earlier than the majority of avian schistosomatid species. Mitochondrial *cox*1 studies, however, provide less consistent results [39,40], possibly due to the saturation of third codon positions [80,81]. Overall, there is a lack of sufficient detail about intermediate and definitive host use patterns and the cercarial anatomy for *Macrobilharzia*. For instance, whereas the cercariae for the M clade member W688 possess eyespots, cercariae within the SB clade do not. It is not known if cercariae of *Macrobilharzia* spp. have eyespots or not.

#### 3.1.5. DAS Clade

Efforts to describe schistosomatid diversity have yielded numerous novel DAS lineages [21,28,30,31,33]. Intermediate host switching is evidently recurring within DAS and can be hypothesized as a probable mechanism of diversification; at least eight intermediate host-switching events have occurred within the DAS clade, at least two of which have been from freshwater to marine environments [21]. Planorbidae species are predominant hosts within DAS, and are associated with *Bilharziella*, *Dendritobilharzia*, Avian Schistosomatid C [31], Avian Schistosomatid D (this study), and *Anserobilharzia* (Figure 3). Caution as to snail host use among early, and likely extinct, representatives of the DAS clade is warranted because representatives of at least six other gastropod families are known to be exploited collectively by extant members of the DAS clade, which are not all represented in Figure 3 [21,29,30].

The monophyly of DAS is consistently supported in published phylogenies (Table 1), including this study. However, this study provided increased resolution within the DAS clade. *Bilharziella polonica* and Avian Schistosomatidae 2 were strongly supported as being basal to DAS. *Bilharziella polonica* infects a broad taxonomic range of water birds (Anseriformes, Ciconiiformes, Gruiformes, Podicipediformes), and this may suggest that ancestors to the DAS + HS clade similarly infected a broad host range, as might be expected during a transition from a marine to freshwater environment. UCE analysis (Figure 1) supports the breakdown of the remaining DAS taxa into three primary clades.

- Clade 1: *Trichobilharzia*, *Allobilharzia* and *Anserobilharzia* form a well-supported sub-clade within DAS, reported exclusively from Anseriformes, predominantly ducks. Published single loci studies do not consistently resolve the node connecting *Trichobilharzia* and *Allobilharzia* + *Anserobilharzia* [28,82,83,84]. This is a logical grouping as this diverse radiation appears to have occurred in Anseriformes [28,83,84], and *Trichobilharzia* demonstrates a strong association with anatids. The diversification of modern Anatidae has been estimated to have occurred between 25–5 mya [85], which might be used as a hard bound for the radiation of *Trichobilharzia.*
- Clade 2: A second sub-clade within DAS is composed of several lineages retaining the names used from previous studies, Avian Schistosomatidae C (C2, C4, and C11 were sampled, [31]). Avian Schistosomatidae C is a complex of several species distributed globally (Americas, Europe, and Africa), none of which have yet been formally named [31].
- Clade 3: *Gigantobilharzia, Dendritobilharzia,* and six undescribed genera form Clade 3. Branch lengths within this clade, specifically the distance between tips, are large, which may suggest missing taxa. Remarkably, the taxa in Clade 3 (Figure 1) are hosted by at least four different snail families (including one marine family) and three orders of birds (Passeriformes, Charadriiformes, and Anseriformes) recovered from Argentina, Kenya, and across North America.

Anseriformes are a common host group throughout DAS and are utilized by species of *Dendritobilharzia*, Avian Schistosomatid A, Avian Schistosomatid C, and Avian Schistosomatid D and *Trichobilharzia*, possibly the most speciose schistosomatid genus [21,28].

#### 3.1.6. HS Clade

UCE analyses provided strong support for a sister relationship between the HS and DAS clades, and accordingly a second independent switch into mammals (as posited by Snyder and Loker [25]). Based on topology (Figure 1 and Figure 2), one can hypothesize that DAS and HS shared a recent common ancestor, likely in what is now North America. *Heterobilharzia* and *Schistosomatium* are both monotypic mammal-infecting genera, exclusive to North America, and are strikingly depauperate relative to extant DAS or SB taxa.

HS may have been historically more speciose, as the loss of the North American mammalian megafauna led to the extinction of many lineages relative to the mammalian megafauna in Africa and Asia [86], which did not experience similar losses. *Schistosomatium douthitti* is a rodent parasite whereas *Heterobilharzia americana* is primarily a raccoon (Procyonidae) and dog (Canidae) parasite, though it has been reported from a broad spectrum of mammals [87,88,89]. Both depend solely on lymnaeid snail hosts. Species-level investigations are required to verify if *H. americana* is truly a single species and not a complex of cryptic species [89]. Correa et al. [90] reconstructed phylogenetic relationships among Lymnaeidae and demonstrated a distinct split among North American and Old World lymnaeids, and an accurate date for this split could provide a hard bound on when the HS clade evolved in North America.

### 3.2. Diversification via Extensive Intermediate Host-Switching

Across the Schistosomatidae, definitive host associations appear less constrained, at the level of host order or below, relative to intermediate host associations [8,91,92]. Our study supports the idea that population isolation via intermediate host-switching mediates schistosomatid speciation [21,93,94]. Relative to schistosomatids, which infect 16 families within Caenogastropoda and Heterobranchia [21], other prominent digenean families engage relatively few gastropod families: for instance, both the Fasciolidae and Paramphistomidae each exploit two gastropod families [95,96]). Schistosomatids are more similar in this regard to their two closely related blood fluke families, the Spirorchiidae and Aporocotylidae. Spirorchiids have been reported from Vetigastropoda, Caenogastropoda, Heterobranchia, and Annelida [97]. Aporocotylids infect Annelida, Bivalva, Caenogastropoda, Heterobranchia, and Neritomorpha [98].

Most obviously within the DAS clade, intermediate host-switching events are characterized by short inter-nodal branches and do not appear to be associated with deep divergence events. This suggests that such host-switches can occur relatively rapidly and frequently over evolutionary time, and genetic differences among lineages (especially for nuclear loci) have not had time to accumulate.

Why intermediate host-switches are seemingly so numerous within Schistosomatidae is an open question, one understandably hard to capture with experimental studies. Some hypotheses are as follows: (1) The *facilitated host-switching hypothesis*—intermediate host-switches may be facilitated by coinfections with other snail parasites [21,99]. For instance, at least some *Austrobilharzia* species seem to specialize in actively exploiting the presence of other trematode larvae to colonize their snail hosts [100], and in some cases prior trematode infections enable schistosomatids to exploit new host species [101]. (2) The *ecological fitting hypothesis* would suggest that schistosomatid larvae may retain pre-adaptations, evolved from ancestral host-use, that enable them to infect different gastropod lineages [5]). The *evolutionary potential hypothesis* (this study) proposes that schistosomatid species maintain high genetic diversity and large effective population sizes, favoring the presence of rare alleles that might confer infectivity in a new gastropod lineage, as observed in population level studies of *Schistosoma japonicum* [92,102,103], *Trichobilharzia* spp. [10], and to a lesser extent *S. mansoni* [103,104,105,106,107]. One expectation may be that schistosomatids of migratory avian hosts have large effective population sizes and high genetic diversity, and therefore adaptive potential [108,109], which may favor switching into new snail hosts [94]. That schistosomatid clades associated with migratory birds have high rates of host switching is likely also related to bird vagility [10,110], which frequently moves parasites to new habitats where they have the opportunity to encounter different snail lineages. For these reasons, one might hypothesize that host-switching is a predominant mode of diversification among parasites obligate to migratory hosts.

### 3.3. Phylogenomic Considerations and Future Directions

#### 3.3.1. Phylogenomics of Schistosomatids

The diminutive size and location of schistosomatids within their hosts (venous or rarely arterial system) present significant challenges to next-generation sequencing applications and downstream phylogenomic analysis. Species of several novel, unnamed genera were not included in this dataset despite considerable effort, due to inadequate specimen quality; their future inclusion may increase resolution still more. We found that, in general, adult worms yielded the most DNA and average number of reads, and the best capture success for multiple loci. Notably, among cercarial samples, none of these measures improved, on average, by increasing the number of cercariae extracted, suggesting this pattern is not solely related to amount of starting tissue.

Modifications of our sequence capture and library preparation protocols (see the Methods section) increased the recovery rate of UCE loci (a three-fold increase). Future studies performing sequence capture on organisms with a low quantity of or degraded museum specimens should consider incorporating these modifications. Large amounts of sequence data are fairly robust to missing data [111], and it has been demonstrated that incomplete taxon sampling is more unfavorable to phylogenetic inference than missing data [112]. However, the extent of missing data within our final alignments prevented species tree estimation [113,114,115] and statistical assessments of incomplete lineage sorting [43], both of which are the logical next steps to furthering our understanding of schistosomatid evolution. Moving forward, it would be advisable to design a new bait kit to specifically target the most common 1500 UCE loci recovered and increase bait tiling density to improve recovery of UCE loci, thereby reducing missing data.

This study supports the utility of Z-chromosome loci for the phylogenetic reconstruction of schistosomatids. Sex-chromosome markers have proven to be valuable phylogenetic tools among vertebrates [115,116], often performing better than autosomal markers in resolving difficult nodes [43]. The schistosomatid Z-chromosome is the homogametic male chromosome (females ZW), and thereby has a lower effective population size, reduced recombination, and faster sorting rate relative to autosomal markers [115]. Sex-chromosome markers have been shown to improve resolution when incomplete lineage sorting is prevalent [43]. As taxon sampling increases, Z-specific loci may prove to be useful markers for future diversification studies. An ideal strategy might be to design a bait set targeting a greater number of Z-specific loci.

Our analyses (Table 4, Supplementary File S1) demonstrate that our bait set contained phylogenetically informative loci across blood flukes, and even across digeneans. We conclude that the application of UCE loci shows promise in resolving higher level taxonomic questions, such as relationships within Diplostomoidea [47,61] or across Digenea [117,118].

#### 3.3.2. Guiding Future Collection Efforts

Additional schistosomatid biodiversity remains to be discovered. Brant and Loker [21] posit the likelihood of undiscovered schistosomatid lineages within marine environments; several studies support this within the AO clade [119,120,121,122]. Surveys of marine gastropod genera such as *Haminoea* [121] and *Siphonaria* [30,35] (Figure 1) have yielded schistosomatids, but these represent more recent divergences rather than early branching members of the AO clade (Figure 3). The recent, surprising discovery of an aporocotylid fish blood fluke from a dugong [123] provides further impetus to search for additional schistosomatids in marine environments.

The long branch lengths between *Macrobilharzia* and Schistosomatidae sp. W688 may be suggestive of missing taxa; unconfirmed species of *Macrobilharzia* have been reported from Africa and Asia [41,124]. *Macrobilharzia macrobilharzia* infects suliform birds, which have not been sampled as extensively as waterfowl, and would be the logical place to continue investigations into *Macrobilharzia*. Another possibility is that proto-M clade members may have once been represented by many more taxa inhabiting what is recognized as an ancient group of birds, which has been in existence since at least the Paleogene, 66–56 mya [76].

## 4. Materials and Methods

### 4.1. Taxon Sampling

Specimens used for this study are vouchered at the Parasites Division, Museum of Southwestern Biology, University of New Mexico, in Albuquerque New Mexico USA. Locality information, host data and museum catalogue numbers are summarized in Table 2 and can be accessed via the Arctos database.

Schistosomatid samples were collected as described in Brant and Loker [28] and Ebbs et al. [10]. Adult and larval schistosomatids (cercariae, sporocysts and miracidia) were collected between 1995–2017, and were preserved in 95% ethanol or RNAlater. Thirteen of the 17 named genera and 11 currently undescribed lineages were included in this study. The undescribed lineages are related to novel genera or species that lack adult specimens or adult fragments with morphological features for discrimination. Most of the specimens used in this UCE study have been included in previously published schistosomatid molecular phylogenies (*28*S, ITS1, *cox*1 markers) and have originated from various collectors over the past two decades. This manuscript retained the taxa designations and/or collector IDs from the original publications (see Table 2) to facilitate the cross comparison of tree topologies and taxon identification.

UCE probes were designed using the *Schistosoma mansoni* genome (described in detail below); the resultant set was used to mine published genomes. Fourteen schistosomatid taxa and five outgroup taxa (Supplementary File S1) were mined for UCE loci and included within this study. The number of recovered loci relates to the phylogenetic similarity of the taxa with *S*. *mansoni* and the quality and completeness of the published SRA (Sequence Read Archive) data.

### 4.2. Sample Preparation

Several extraction protocols were used to obtain template DNA of sufficient quantity and quality, which is often a limiting factor in the number and quality of the UCE loci recovered from museum specimens [125,126]. Most samples were extracted using silica-based minipreps from either the QIAamp DNA Micro Kit (Qiagen), or the DNeasy Blood and Tissue kit (Qiagen). The QIAamp DNA Micro Kit, which is optimized for small amounts of tissue, performed best across schistosomatid samples. Sample DNA was quantified using Qubit Fluorometric quantification (Thermo Fisher, Waltham, MA, USA) using manufacturer buffers and protocols. Samples selected for sequence capture yielded >0.005–0.5 micrograms of DNA. Sample quality was assessed using a Bioanalyzer (Aligent 2100), however the DNA quantity was generally too low to accurately gauge the fragment size, which varied across samples, with some samples showing significant amounts of degradation and others relatively little. To prevent further degradation, vortexing, sonication, and the thawing/re-freezing of samples was minimized during handling and DNA extraction.

### 4.3. Sequence Capture of Ultra-Conserved Elements

In total, 40 samples were selected for targeted sequence capture of UCEs (Table 2 and Table 3), specifically to address relationships within the Schistosomatidae and to resolve deeper nodes that have evaded phylogenetic placement in previous studies (Table 1). A custom bait set (18,550 baits, 120 nucleotides in length, 2× tiling density) was designed using the *Schistosoma mansoni* genome as a reference. Approximately 4000 UCE loci were targeted. The bait set was manufactured by Arbor Biosciences (www.arborbiosci.com, accessed on 16 February 2017).

Library enrichment procedures for the MYcroarray MYBaits kit (MYbaits^®^ Manual v. 3.02) were followed, but with several modifications to the standard sequence capture and library preparation protocols to accommodate low amounts of DNA and a variable fragment size. To optimize sample preparation protocols, our samples were divided into four distinct sequence capture experiments and sequencing runs (R1, R2, R3 and R4). R1 used standard sticky-end library preparation coupled with standard amplification polymerase. R2–R4 employed blunt end library preparation chemistry and an uracil non-stalling amplification polymerase. This step aimed to reduce adapter dimers, which were abundant in R1 samples. For all runs, a size-selection step following library preparation was not performed due to low DNA quantity. Between R1 and R2–R4, hybridization temperatures were modified (62 or 65 °C, respectively). Post-capture libraries were amplified for 12 cycles. Sequencing of paired-end, 100 bp reads was conducted on an Illumina HiSeq 2000. All sequence data for taxa listed in Table 2 have been made available on NCBI GenBank.

### 4.4. Processing and Alignment of UCE Data

Quality control of the raw reads included trimming adapter contamination and low-quality bases from reads, using the program Trimmomatic [127] and a 4-bp sliding window, quality score 20, and 36-bp minimum read length. Clean reads were processed following the PHYLUCE software package [128], including contig assembly using Trinity [129]. Assembled contigs were then matched to the probe set using lastz [130]. Previously published genomes were downloaded from the NCBI ftp site. For species lacking assembled draft genomes, but for which data were available, we downloaded the Sequence Read Archive (SRA) (https://www.ncbi.nlm.nih.gov/pmc/articles/PMC3013647/, accessed on 30 January 2017) using the NCBI SRA-toolkit (https://trace.ncbi.nlm.nih.gov/Traces/sra/sra.cgi?view=toolkit_doc, accessed on 30 January 2017) in fastq format (fastq-dump in SRA toolkit) and trimmed the data following the steps described above. Draft genome sequences were assembled using the MEGAHIT program [131]. UCE loci were mined from genome sequences following the PHYLUCE protocol. Samples with low capture success, where fewer than 100 UCE loci were recovered, were excluded from subsequent analyses. Alignments were created using MAFFT v. 2.2.7 [132]. Among the outgroup taxa mined for UCE loci (Table 3), the *Echinostoma caproni* genome (PRJEB1207) had the highest number of shared loci and was selected as the outgroup.

All the shared UCE loci for Schistosomatoidea + outgroup (*n* = 47,554 loci, 4,780,079 bases) were aligned for the purposes of phylogenetic reconstruction (Figure 1). A second dataset was limited to loci located on the schistosomatid sex chromosome, the Z-chromosome (*n* = 35, 85 loci, 937,745 bases). Sex chromosomes, due to their reduced recombination rates and effective sizes, have been shown to make excellent phylogenetic markers, often resolving nodes that autosomal loci fail to resolve [43]. Based on our UCE probe design, 139 UCE loci were mapped to the *Schistosoma mansoni* Z-chromosome (PHYLUCE [50]). Of the 139 recovered, 85 loci were shared among ≥70% of the sampled taxa and were aligned for phylogenetic reconstruction. For all datasets, loci were concatenated into a single supermatrix [50,128,132,133].

### 4.5. Alignment Building and Phylogenetic Reconstruction

All alignments were unpartitioned and analyzed in RAxML v.8.0.19 [60] using the GTRGAMMA model of evolution, with 500 thorough bootstrap replicates [50,56] for maximum likelihood analysis. Bayesian inference analysis was performed in BEAST 2 [134] consisting of two replicated runs with four Markov chain Monte Carlo (MCMC) chains, as well as one cold and three heated chains. Each analysis ran for 10,000,000 generations and was sampled every 1000 generations. Likelihood parameters and convergence between runs were assessed using the program Tracer v.1.6 [135] based on EES values greater than 200. The first 2500 trees from each analysis were discarded as burnin. The resulting phylogenetic trees were visualized and manipulated using Fig Tree v. 1.4.4 (https://github.com/rambaut/figtree/ accessed on 30 January 2017).

## 5. Conclusions

Through the analysis of 554 nuclear UCE loci, and a subset of 85 Z-chromosome specific UCE loci, we were able to resolve many pivotal interrelationships within Schistosomatidae, representing the most comprehensive family-level phylogeny to date. Some nodes failed to be resolved or were weakly supported. Further resolution of the two primary radiations (SB + M and DAS + HS) resulting in derived schistosomatid diversity, may be challenging for two possible reasons. (1) Contemporary lineages might have radiated simultaneously and rapidly, resulting in incomplete lineage sorting [42], which could have led to a hard polytomy [136,137]. As mentioned previously, population studies on members of both primary radiations suggest high genetic diversity and effective population sizes, both of which would contribute to incomplete lineage sorting [42,138,139]. (2) Colonization–extinction dynamics are common within parasites [1], and contemporary lineages might be more closely related to one or many extinct lineages than to their most closely related extant taxa. A combination of these factors might work in concert to make further resolution of Schistosomatidae, and potentially other helminth taxa, difficult. Nevertheless, a remarkable history of intermediate host-switching is evident, and indicative of a role as a primary driver of schistosomatid diversification.

## Figures and Tables

**Table 1:** Resolution of pivotal nodes from selected published molecular phylogenies of Schistosomatidae. AO = *Austrobilharzia* + *Ornitobilharzia*; SB = *Schistosoma* + *Bivitellobilharzia*; M = *Macrobilharzia*; DAS = derived avian schistosomatids (*Anserobilharzia*, *Allobilharzia*, *Bilharziella*, *Dendritobilharzia*, *Gigantobilharzia*, *Riverabilharzia*, *Trichobilharzia*, *Marinabilharzia*, *Nasusbilharzia*); HS = *Heterobilharzia* + *Schistosomatium*; N = Clade not recovered/resolved; Y = Clade recovered/resolved; ns = not sampled; partial = weak statistical support or discordance between nuclear and mitochondrial markers. Publications were selected based on having sampled the primary clades of interest.

Source	AO	SB	SB + M	DAS + SB	DAS	DAS + HS	HS	Loci
[25]	N	ns	ns	ns	Y	partial	Y	*28*S
[26]	N	ns	ns	N	Y	N	Y	*18*S, *28*S + *cox*1
[27]	Y	Y	N	partial	partial	partial	Y	*18*S, *28*S + *cox*1
[28]	Y	N	N	N	Y	partial	N	*18*S, *28*S, ITS + *cox*1
[37]	ns	Y	Ns	ns	ns	Ns	ns	*18*S, *28*S + *cox*1
[21]	Y	ns	Ns	Y	Y	N	Y	*18*S, *28*S, ITS + *cox*1
[38]	N	partial	N	N	Y	N	Y	*18*S, *28*S + *cox*1
[34]	ns	Y	Y	Y	N	N	Y	*28*S
[35]	Y	N	N	N	Y	N	Y	*28*S, ITS + *cox*1
[39]	Y	partial	N	Y	Y	partial	Y	*28*S + *cox*1
[40]	Y	partial	N	partial	Y	partial	Y	*28*S + *cox*1

**Table 2:** The included taxa and relevant accession information. Sample ID is a unique identifier given by the collector. The complete spatio-temporal data can be accessed through the Parasites Division, Museum of Southwestern Biology host and or/parasite records maintained in Arctos. CAN = Canada, USA = United States of America, UA = Ukraine, NP = Nepal, ZA = South Africa, AR = Argentina, BR = Brazil.

Taxa	Sample ID	Host	Locality	NCBI	MSB:Para:#
*Anserobilharzia brantae*	W335	*Gyraulus parvus*	CAN	SRR19593566	14745
	W351	*Branta canadensis*	CAN	SRR19593565	7984
	W333	*Gyraulus parvus*	CAN	SRR19593554	14744
*Austrobilharzia variglandis*	SAL63.81	*Larus delawarensis*	CAN	SRR19593543	32451
	SAL63.80	*Larus delawarensis*	CAN	SRR19593533	29053
*Bilharziella polonica*	W930	*Anas platyrhynchos*	UA	SRR19593532	32667
*Bivitellobilharzia nairi*	W465.2	*Rhinoceros unicornis*	NP	SRR19593531	29075
*Dendritobilharzia pulverulenta*	W926	*Anas crecca*	USA	SRR19593530	29034
	W836	*Anas discors*	USA	SRR19593529	20795
*Gigantobilharzia huronensis*	W414/513	*Physa* sp.	USA	SRR19593528	18687/25488
	W678	*Physa* sp.	USA	SRR19593564	29074
*Heterobilharzia americana*	W805	*Procyon lotor*	USA	SRR19593563	19286
*Macrobilharzia macrobilharzia*	W931	*Anhinga anhinga*	USA	SRR19593562	32668
*Schistosomatium douthitti*	SAL95_60	*Stagnicola* sp.	CAN	SRR19593561	2861
*Schistosoma bovis*	PM1	*Bulinus* sp.	KE	SRR19593560	32666
*Schistosoma nasalae*	W546	*Indoplanorbis exustus*	NP	SRR19593559	—
*Schistosoma spindale*	W545	*Indoplanorbis exustus*	NP	SRR19593559	—
*Schistosoma edwardiense*	W957	*Biomphalaria* sp.	KE	SRR19593557	32609
*Schistosoma* sp.	W555	*Indoplanorbis* sp.	NP	SRR19593556	—
*Ornithobilharzia canaliculata*	W393	*Larus occidentalis*	USA	SRR19593555	18542
*Trichobilharzia querquedulae*	W664	*Spatula smithii*	ZA	SRR19593553	19000
*Trichobilharzia stagnicolae*	W233	*Stagnicola emarginata*	USA	SRR19593552	19509
*Trichobilharzia* cf. *szidati*	W620A	*Lymnaea stagnalis*	USA	SRR19593551	259
Avian Schistosomatidae sp. A	W613	*Melanitta deglandi*	USA	SRR19593550	25264
Avian Schistosomatidae lineage 2	W634	*Chilina perrieri*	AR	SRR19593549	7969
Avian Schistosomatidae sp. B	W399	*Physa acuta*	USA	SRR19593548	18677
Avian Schistosomatidae sp. C2	W402	*Gyraulus parvus*	USA	SRR19593547	18680
Avian Schistosomatidae sp. C4	W847	*Biomphalaria glabrata*	BR	SRR19593546	25514
Avian Schistosomatidae sp. C11	W607	*Anas americana*	USA	SRR19593545	25258
Avian Schistosomatidae sp. C11	W616A	*Gyraulus* sp.	USA	SRR19593544	19650
Avian Schistosomatidae sp. D	W342	*Gyraulus parvus*	CAN	SRR19593542	18619
Avian Schistosomatidae sp. G	W877	*Ceratophallus* sp.	KE	SRR19593541	32612
*Marinabilharzia patagonense*	W637A	*Siphonaria lessoni*	AR	SRR19593540	18935
Avian Schistosomatidae lineage 3	C1	*Chilina neuquenensi*	AR	SRR19593539	7970
Avian Schistosomatidae sp. M1	W216	*Haminoea japonica*	USA	SRR19593538	18660
Schistosomatidae sp. W688	W688	*Indoplanorbis exustus*	NP	SRR19593537	18710
**Outgroups**					
*Vasotrema* cf. *robustum*	W411	*Physa* sp.	USA	SRR19593536	18690
*Vasotrema* cf. *robustum*	PS	*Physa* sp.	USA	SRR19593535	18715
Spirorchiidae	W702	*Biomphalaria straminea*	BR	SRR19593534	20804
**Published Genomes**					
*Allobilharzia visceralis*				SAMEA2201407	
*Schistosoma hematobium*					
*Schistosoma mansoni*					
*Schistosoma japonicum*					
*Schistosomatium douthitti*				SAMEA1920831	
*Trichobilharzia szidati*				PRJEB461	
*Trichobilharzia regenti*				SAMEA2422295	
*Echinostoma caproni*				PRJEB127	

**Table 3:** Summary of sequence capture data. Sample IDs separated with an (/) indicate that samples were pooled to increase DNA quantities; in these samples DNA (µg) is additive. The Schistosomatid life stage is indicated as A (adult), M (miracidia) or C (cercariae). nq = not quantified.

Taxa	Sample ID	Life Stage	DNA [µg]	No. of Contigs	No. of bp	UCEs
**Schistosomatidae**						
*Anserobilharzia brantae*	W352/W340	C	0.562	1158	330,978	48
	W335	C	nq	16,743	4,870,006	1334
	W351	C	>0.005	5907	1,836,149	551
*Austrobilharzia variglandis*	W396/359	A	0.0352	145	35,487	6
	W697	A	0.0193	8344	2,495,016	1440
*Bilharziella polonica*	W930	A	0.0155	17,081	5,897,771	1677
*Bivitellobilharzia nairi*	W465	M	0.078	18,455	5,052,402	1336
	BN2011	M	nq	3696	1,162,332	47
*Dendritobilharzia pulverulenta*	W836	A	nq	26,978	8,405,837	1737
	W926	A	0.0944	47,498	14,794,680	1631
*Gigantobilharzia huronensis*	W414/513	C	0.0375	242,326	72,500,699	1650
	W678	C	0.0325	48,623	16,046,122	1582
*Heterobilharzia americana*	W805	A	0.133	442,043	139,396,852	1784
*Macrobilharzia macrobilharzia*	W931	A	0.0472	3508	1,580,643	1590
*Marinabilharzia patagonense*	W637A	C	0.163	34,871	13,736,793	1749
*Ornithobilharzia canaliculata*	W393	A	0.15	31,569	8,873,305	1441
*Schistosomatium douthitti*	SAL95.60	C	nq	15,755	4,482,586	597
*Schistosoma bovis*	PM1	C	nq	6316	4,181,809	1873
*Schistosoma nasale*	W546	C	0.0107	41,764	14,799,490	1830
*Schistosoma spindale*	W545	C	0.0062	15,206	5,394,821	1885
*Schistosoma edwardiense*	W957	C	nq	21210	8,383,839	1800
*Schistosoma* sp.	W555	C	0.1455	15,865	7,245,729	1862
*Trichobilharzia querquedulae*	W929	A	0.0224	70,532	21,183,008	1576
*Trichobilharzia stagnicolae*	W233	C	0.0531	18,675	13,662	658
*Trichobilharzia* cf. *szidati*	W620A	C	nq	48,011	15,967,522	1123
Avian Schistosomatidae sp. A	W613	A	0.091	10,458	3,571,057	1511
Avian Schistosomatidae sp. B	W399	C	0.0905	9349	2,995,586	1115
Avian Schistosomatidae sp. C2	W402	C	0.0845	15,168	4,939,724	849
Avian Schistosomatidae sp. C4	W847	C	nq	35,118	10,814,130	1732
Avian Schistosomatidae sp. C11	W607	C	0.265	34,656	53,303	1599
Avian Schistosomatidae sp. C11	W616A	C	0.084	32,003	10,790,620	1409
Avian Schistosomatidae sp. D	W342	C	0.43	13,126	3,804,186	171
Avian Schistosomatidae sp. G	W877	C	nq	1227	356,239	330
Avian Schistosomatidae lineage 2	W634	C	nq	6368	1,553,874	419
Avian Schistosomatidae lineage 3	C1	C	nq	2576	764,111	458
Schistosomatidae sp. W688	W688	C	0.0337	8006	3,890,904	1428
**Non-Schistosomatidae**						
*Vasotrema* cf. *robustum*	W411	C	nq	17,681	4,449,659	372
Spirorchiidae	W702	C	nq	47,339	15,477,172	885
*Vasotrema* cf. *robustum*	PS	C	nq	41,958	12,949,767	473

**Table 4:** Average nucleotide similarity (%) between Schistosomatidae, Spirorchiidae and Aporocotylidae for 25 UCE loci.

	Spirorchiidae	Aporocotylidae
*Trichobilharzia regenti*	84.90	80.24
*Heterobilharzia americana*	84.30	79.50
*Schistosoma mansoni*	83.60	80.84
*Macrobilharzia macrobilharzia*	85.13	80.48
AO Clade	83.40	80.56

## Data Availability

The data presented in this study are openly available on the NCBI Sequence Read Archive.

## Supplementary Materials

The following supporting information can be downloaded at: https://www.mdpi.com/article/10.3390/pathogens11070769/s1, Supplementary File S1: Table of all published digenean genomes mined for UCE loci; Supplementary File S2: Likelihood scores of RAxML phylogenies with varying levels of missing data; Supplementary File S3: Excel file of the presence and absences of the 554 UCE loci by taxa; Supplementary File S4: Excel file of the presence and absences of the 85 Z-chromosome specific UCE loci by taxa.
