# Supplementary material for: Phylogenomics and Diversification of the Schistosomatidae Based on Targeted Sequence Capture of Ultra-Conserved Elements
Source: Pathogens. 2022 Jul 5;11(7):769. doi: 10.3390/pathogens11070769 (PMC9321907; doi:10.3390/pathogens11070769)
Supplement: Supplementary file 1 [file pathogens-11-00769-s001.zip › Ebbs2022_UCE_Additional File 2.pdf]

Additional File S2. Summary of UCE Supermatrix alignments.

Alignment	% missing data	No. Loci	No. bp	-logLN
1	18.19%	113	41615	-249,913.44
2	21.22%	258	95215	-552,102.19
3	24.25%	470	167917	-958,990.67
4	27.28%	756	265282	-1,487,900.85
5	30.31%	1,070	372577	-2,061,422.86
6	33.34%	1,364	474081	-2581373.332
7	39.40%	1,819	627118	-3350563.131
8	42.43%	1,970	627118	-3586512.511
9	48.90%	2,006	677378	-3809408.073
10	51.50%	2,082	736137	-3841912.875
11	54.55%	2,144	743745	-3866958.204
12	60.60%	2,159	753058	-3903325.447
13	63.60%	2,179	755774	-3912154.447
