# Supplementary material for: Phylogenomics and Diversification of the Schistosomatidae Based on Targeted Sequence Capture of Ultra-Conserved Elements
Source: Pathogens. 2022 Jul 5;11(7):769. doi: 10.3390/pathogens11070769 (PMC9321907; doi:10.3390/pathogens11070769)
Supplement: Supplementary file 1 [file pathogens-11-00769-s001.zip › Ebbs2022_UCE_AdditionalFile1.pdf]

Additional File S1. Published genomes mined for UCE loci.

Sample ID	Taxa	UCE Loci
**Schistosomatidae**		
SAMEA2201407	*Allobilharzia visceralis*	1254
PRJEB519	*Schistosoma curassoni*	2421
PRJNA273970	*Schistosoma haematobium*	2423
PRJEB2425	*Schistosoma haematobium*	2422
PRJNA354903	*Schistosoma japonicum*	2384
PRJNA286685	*Schistosoma japonicum*	2382
PRJEB13625	*Schistosoma mansoni*	2417
PRJNA302212	*Schistosoma mansoni*	2411
PRJEB523	*Schistosoma mattheei*	2422
PRJEB522	*Schistosoma margrebowiei*	2421
PRJEB526	*Schistosoma rodhaini*	2424
SAMEA1920831	*Schistosomatium douthitti*	115
SAMEA2422295	*Trichobilharzia regenti*	2400
PRJEB4661	*Trichobilharzia szidati*	1219
**Out-groups**		
PRJEB3954	*Dicrocoelium dendriticum*	58
PRJEB1207	*Echinostoma caproni*	296
PRJNA438721	*Fasciola gigantica*	260
PRJNA392273	*Opisthorchis viverrini*	214
PRJNA248332	*Paragonimus westermani*	246
